# 3D geometric morphometric analysis of morphological covariation in the modern human postcanine dentition

**DOI:** 10.1038/s41598-026-56919-2

**Published:** 2026-06-09

**Authors:** Petra G. Šimková, Viktoria A. Krenn, Cinzia Fornai, Lisa Wurm, Vanda Halász, Dominika Lidinsky, Gerhard W. Weber

**Affiliations:** 1 Department of Evolutionary Anthropology, University of Vienna, Vienna, Austria; 2 Human Evolution and Archaeological Sciences (HEAS), University of Vienna, Vienna, Austria; 3 Institute of Evolutionary Medicine, University of Zurich, Zurich, Switzerland; 4 Fraunhofer Austria Research GmbH, Klagenfurt, Austria; 5 Department of Research in Occlusion Medicine, Vienna School of Interdisciplinary Dentistry – VieSID, Klosterneuburg, Austria; 6 Center for Clinical Research, University Clinic of Dentistry Vienna, Medical University of Vienna, Vienna, Austria; 7 Medical Technology Cluster, Business Upper Austria – OÖ Wirtschaftsagentur GmbH, Linz, Austria; 8 LearnChamp Consulting GmbH & Co KG, Vienna, Austria; 9 Sandoz GmbH, Kundl, Austria; 10 Core Facility for Micro- Computed Tomography, University of Vienna, Vienna, Austria

**Keywords:** Premolars, Molars, Geometric morphometrics, Morphological correlation, 3D shape analysis, Anatomy, Evolution, Health care

## Abstract

Analysis of 3D shape covariation patterns between different tooth types is rarely investigated but is important for human evolution, dental anthropology, and medicine. We explored shape covariation of the postcanine dentition (excluding third molars) within and between dental arches using geometric morphometrics. Based on micro-CT scans of 526 teeth from 136 individuals, we analyzed dentine crowns, considering enamel–dentine junction (EDJ) occlusal surface and cervical outlines. High pairwise correlations occurred within dental arches (lower P3–P4, r₁ = 0.89; upper P3–P4, r₁ = 0.81; upper M1–M2, r₁ = 0.86). Between antagonists, correlations varied from the highest in upper–lower M1 (*r* = 0.90) to the lowest in upper P4–lower M1 (*r* = 0.58). Unexpectedly high covariation appeared between some non-occluding teeth (lower P3–upper M2, r₁ = 0.88). Relatively high covariation also occurred between different tooth classes serving similar masticatory functions: lower P4–upper M1 (r₁ = 0.79) and upper P4–lower M1 (r₁ = 0.77). Crown height and relative proportions emerged as the main factors driving covariation within the postcanine dentition, independently from allometric effects. Overall, morphological integration is stronger within the same dental arch than between antagonistic teeth. These results provide baseline data for developmental, phylogenetic, and functional studies of the human postcanine dentition, including potential phenotype–genotype associations.

**Supplementary Information:**

The online version contains supplementary material available at 10.1038/s41598-026-56919-2.

## Introduction

Human dental morphology is a topic of interest in the fields of dental medicine, biological anthropology, and human evolution, which has lately evolved into the exploration of 3D dental geometry^1–16^. Shape covariation of human postcanine teeth is still largely unexplored, yet it represents fundamental knowledge for the understanding of the human masticatory system and its development.

Quantitative genetics distinguishes population phenotypic variation effects into genetic, non-genetic, and variation due to covariate effects. Similarly, phenotypic correlations can be broken down into genetic and non-genetic mechanisms, enabling the exploration of genetic covariation patterns underlying phenotypic traits^17^. Understanding the sources of phenotypic covariation, however, requires integrating approaches with insights into the biological processes that generate and constrain trait variation.

The development of anatomical structures and traits is a coordinated process driven by growth factors, induction processes, signaling molecules and cascades, in which neighboring elements interact with each other to create tightly integrated forms. This process, referred to as developmental integration, orchestrates the formation of various tissues, generating cohesive patterns of phenotypic variation throughout growth. Apart from developmental integration, pleiotropy (genes affecting multiple traits^18–21^ as well as linkage disequilibrium (deterministic association of alleles on multiple loci, with an effect on various traits^22^ affect phenotypic covariation. Morphological integration may further reinforce these effects as it results from physical interdependence of parts through shared architecture or mechanical interrelation, and contributes to patterns of covariation, particularly in highly integrated anatomical systems^23^. Functional integration leads to correlational selection that can result in linkage disequilibrium and induce covariation between structures that are not developmentally linked. However, even if given strong selective pressure, the effect of linkage disequilibrium on phenotypic covariation seems to be low compared to the influence of developmental integration and pleiotropic effects^22^.

Developmental genetic studies have contributed to the understanding of the association between dental genotype and phenotype^17,24^. However, although the odontogenetic morphological stages are well-documented^25^, the genetic pathways regulating odontogenesis still need elucidation. More than 2400 genes appear to be involved in odontogenesis^26,27^, with the majority of the related proteins belonging to four signaling pathway families; BMP, FGF, SHH, and WNT^28–32^, as well as to proteoglycans^33^. Various evolutionary developmental models have been proposed to explain odontogenesis^34,35^. Kavanagh et al^36^. put forward the inhibitory cascade model, which describes how the development of one tooth might influence the development of the adjacent teeth, demonstrating that activator-inhibitor dynamics determine size differences between molars and significantly contribute to metameric variation within the dental row, as it can be used to explain morphological reduction in distal molars^10^. A high genetic influence on human dental morphology has been shown in twin studies^37,38^. In an experimental study on *Cryptotis parva’s* molars^39^, narrow-sense heritability (namely, the proportion of total phenotypic variance that is attributed to additive genetic variance alone) of overall crown shape has been determined at h^2^ = 0.34 (in a range between 0 and 1), reflecting the important contribution of the genetic makeup in determining the shape of the tooth crown in shrews.

Because of functional constraints and efficient mastication, we expect the shape of opposing teeth (antagonists^40^ to show higher covariation than other tooth pairs. However, since current knowledge of odontogenetic mechanisms and modular organization of teeth with significant genetic covariation within jaws^19,41^ suggests even higher shape covariation within dental arches, we expect to find the strongest covariation there.

Building upon the knowledge from previous genetic and developmental studies, as well as metameric variation and integration studies^10,42^, we want to contribute to the understanding of morphological integration patterns in the modern human postcanine dentition using phenotypic data. We did so by exploring shape covariation using landmark based 3D geometric morphometric approach, working on µCT data of a heterogenous modern human sample. We focused on the postcanine dentition exploring the dentine crowns of upper and lower third and fourth premolars (P3, P4) and first and second molars (M1, M2). This approach does not allow for comprehensive exploration of the covariation processes within the postcanine dentition, as the morphology of the dental crowns represents the end product of the interplay of genetic, epigenetic, and developmental processes. Unraveling the full scope and nature of these mechanisms therefore requires integration of extensive datasets, including a detailed understanding of the phenotype examined in this study.

## Results

### Shape covariation of the dentine crown

For the dentine crown dataset, the pairwise correlations and percentages of total covariance according to the Singular Warp Score 1 are reported in Tables 1 and 2 and Supplementary Tables S2 and S3, respectively. Integration patterns were first assessed using the original Procrustes coordinates to capture the total morphological covariation. Subsequently, 2-block Partial Least Squares analysis (2B-PLS) was applied also to the multivariate residuals of a Procrustes regression on size to investigate the effect of allometry on the covariation pattern between postcanine teeth.

**Table 1:**
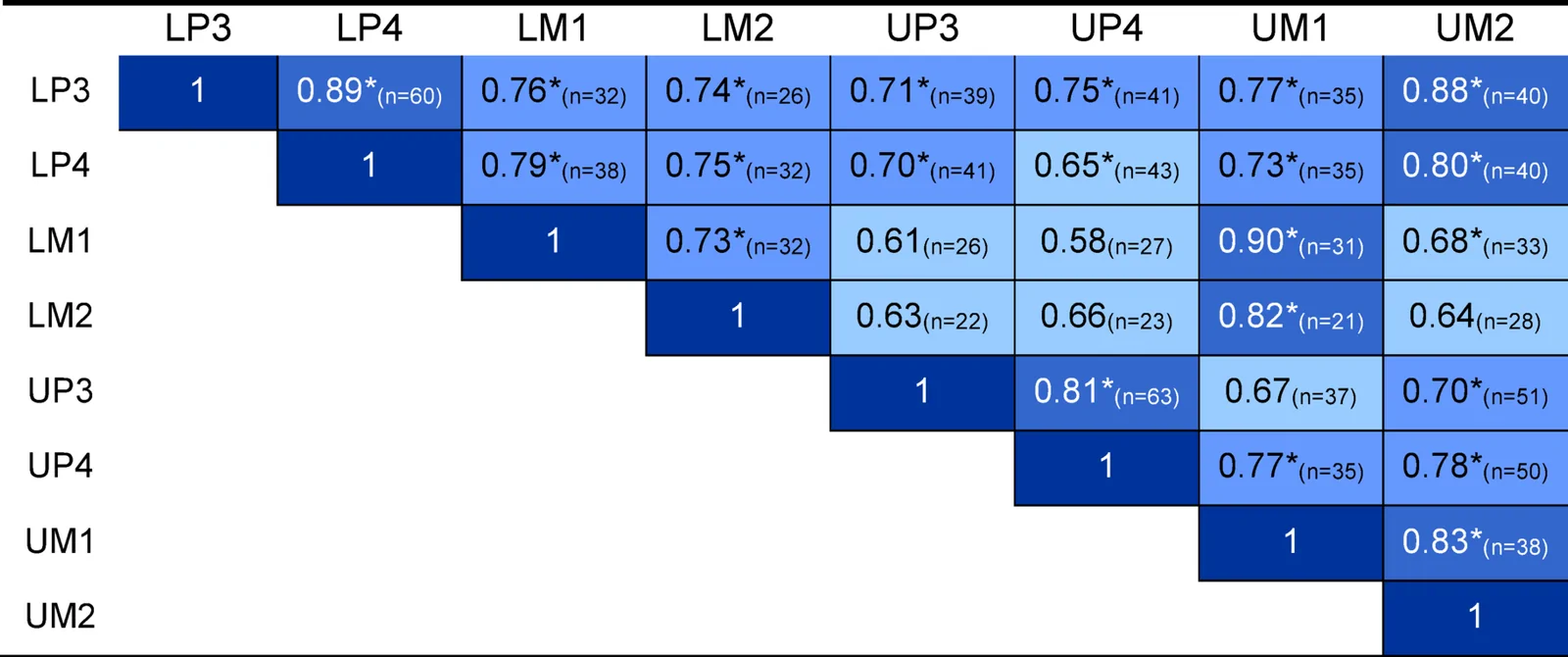
Pairwise correlation values resulting from 2-block Partial Least Squares Analyses of the dentine crown datasets. (L = lower; U = upper; P3 = third premolars: P4 = fourth premolars; M1 = first molars; M2 = second molars); * marks significant results.

The pairwise correlation values ranged from r_1_ = 0.55 to r_1_ = 0.90. As there are no standardized thresholds for interpreting correlation coefficients that apply universally across different types of data^43^, we referred to the obtained values within the specific context of this study and the data distribution observed on our sample. The range of our empiric correlation values was categorically divided, referred to as very high (> 0.79), high (0.79–0.69) and moderate (< 0.69) pairwise correlation, for easier interpretation and discussion of the results. This classification is anchored by the coefficient of determination, specifically, the 0.69 threshold represents the point where approximately half of the morphological variance is shared between blocks (r^2^ ≈ 0.48)^44^, while the grand median of our total dataset (0.73) is encompassed in the high category. As the accuracy of the observed pairwise correlation values is determined by their effect sizes, they should be interpreted as such.

**Fig. 1:**
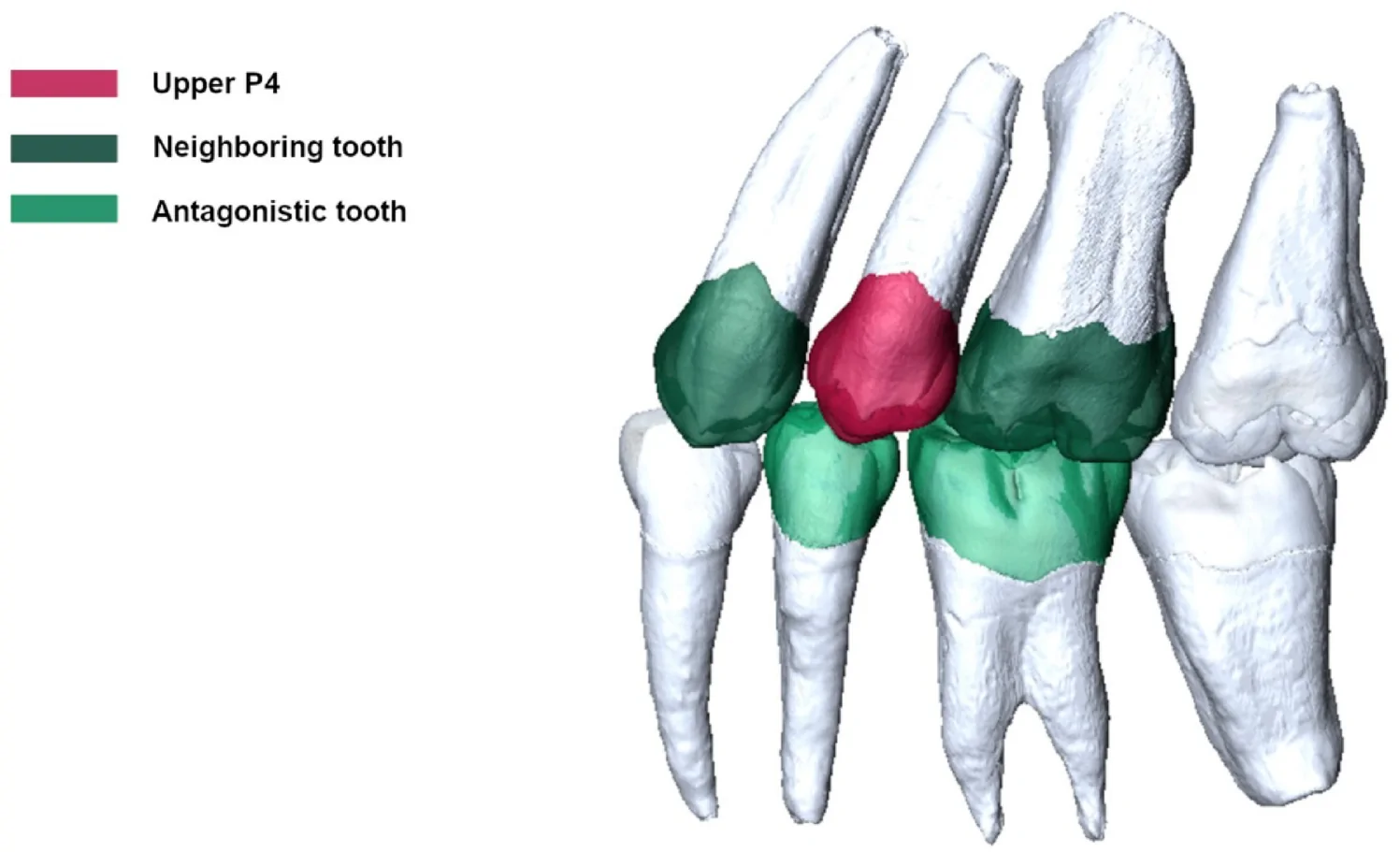
The postcanine dentition (third molars excluded) in occlusion. The color coding is used to describe the specific relationship of the upper fourth premolar to its neighboring and antagonistic teeth.

When analyzing the original shape data, we found high to very high pairwise correlation (Table 1) in all pairs of the same tooth class (thus either premolars or molars) within the dental arch (lower P3 and P4, r_1_ = 0.89; upper P3 and P4, r_1_ = 0.81; upper M1 and M2, r_1_ = 0.83, lower M1 and M2, r_1_ = 0.73). The correlation values between antagonists (Fig. 1) varied notably from very high − obtained for the pair upper/lower M1 (r_1_ = 0.90) and upper M1 and lower M2 (r_1_ = 0.82), to high − in upper/lower P3 (r_1_ = 0.71), lower P4 and upper P3 (r_1_ = 0.70), and moderate in upper/lower P4 (r_1_ = 0.65), upper/lower M2 (r_1_ = 0.64), and in upper P4 and lower M1 (r_1_ = 0.58). High to moderate correlation values were obtained from non-neighboring tooth types within the same jaw, as well as tooth types that do not articulate in normal occlusion. However, unexpectedly high values were detected in some non-neighboring and non-occluding tooth type pairs, all including a lower premolar (lower P3 and M1, r_1_ = 0.76; lower P3 and upper P4, r_1_ = 0.75; lower P3 and upper M1, r_1_ = 0.77; lower P3 and upper M2, r_1_ = 0.88; lower P4 and M2, r_1_ = 0.75; lower P4 and upper M2 r_1_ = 0.80). A high pairwise correlation was also found between lower P4 and M1 (r_1_ = 0.79), and upper P4 and M1 (r_1_ = 0.77), which are the only pairs formed by adjacent teeth belonging to different dental classes yet serving similar functional purposes during mastication^45^. Additionally, the correlation between upper P4 and M2 (r_1_ = 0.78) was as strong as between upper P4 and M1.

The analysis of the multivariate residuals of a Procrustes regression on size showed that the majority of observed correlation values remained unchanged, with the maximum decrease in correlation value limited to 0.06 (Table 2, Supplementary Table S3). A substantial drop in correlation values (> 0.09) was observed in tooth pairs of lower premolars with lower M2 and upper M1 (lower P3 and M2, r1 = 0.57; lower P4 and M2, r1 = 0.58; lower P3 and upper M1, r1 = 0.67). The results were supported by a decrease in effect size observed in these analyses (Supplementary Table S3).

**Table 2:**
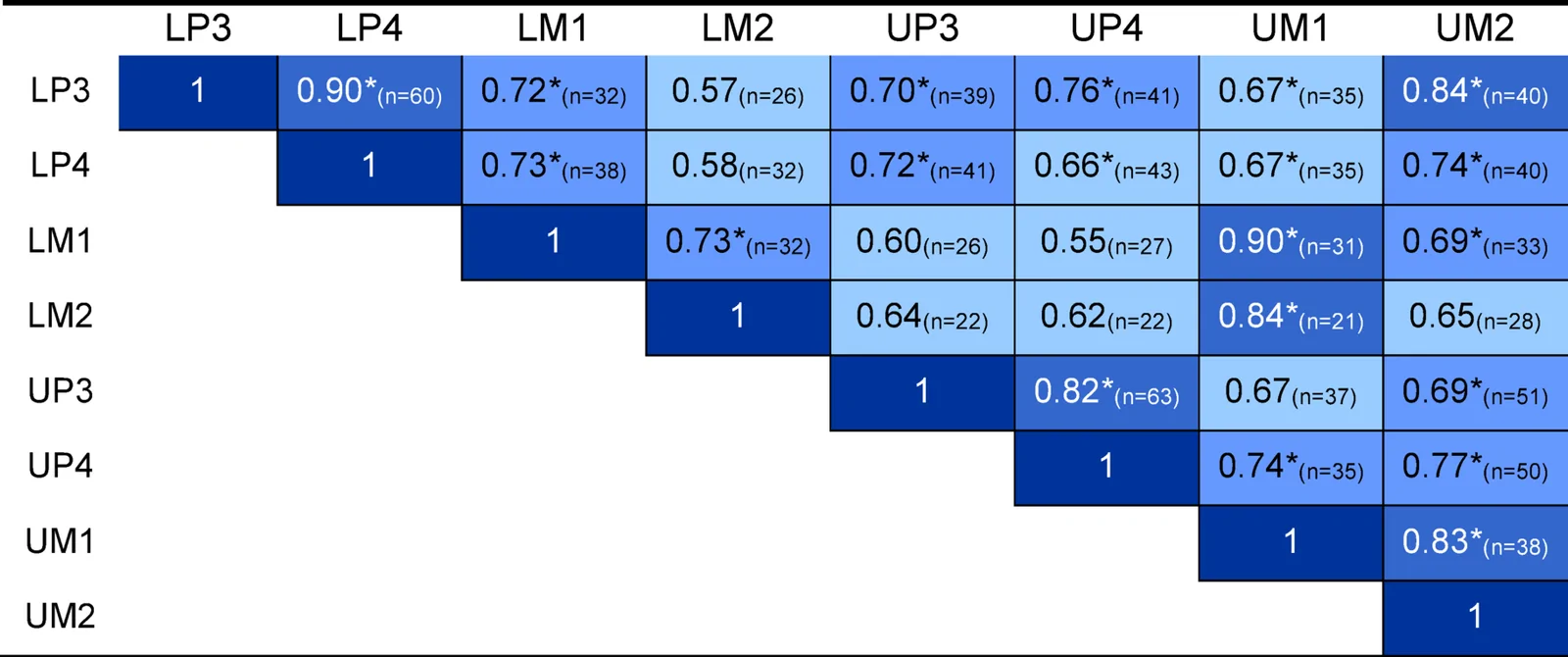
Pairwise correlation values resulting from 2-block Partial Least Squares Analyses of the dentine crown multivariate residuals datasets. (L = lower; U = upper; P3 = third premolars: P4 = fourth premolars; M1 = first molars; M2 = second molars); * marks significant results.

Statistical significance of the results of all 2B-PLS analyses was determined using permutation tests. For each tooth pair analysis, one block was randomly shuffled across specimens 10,000 times to generate a null distribution of the r_1_ statistic. Reported correlations were proved significant except between upper premolars and lower molars, as well as in the pairs of upper P4 and upper M1, and between upper and lower M2.

**Fig. 2:**
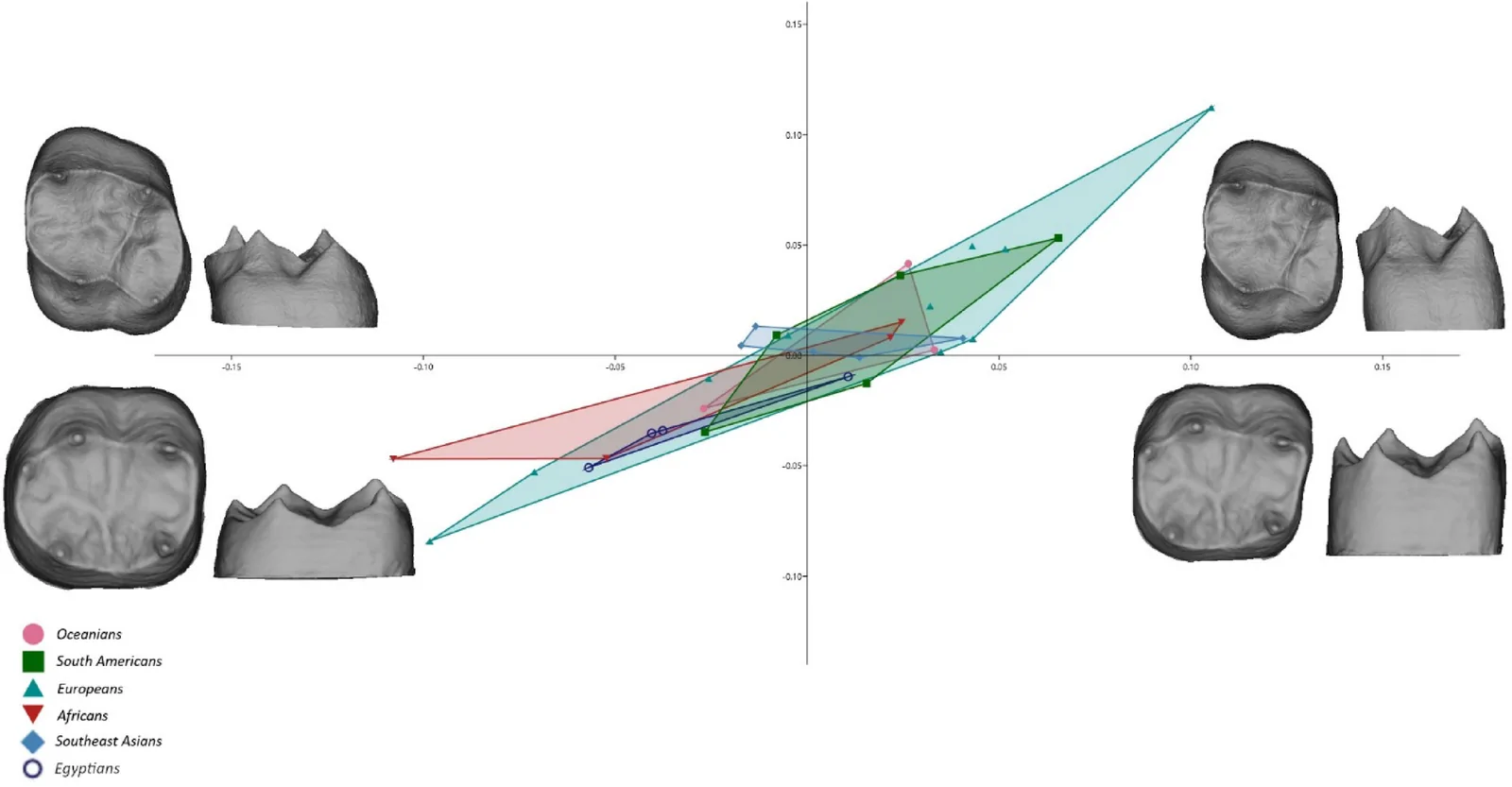
2B-PLS plot for the covariation of upper and lower first molars’ dentine crowns; the warping shows the actual shape variation in occlusal and lingual view at the extremes of the range of distribution along the Singular Warp scores 1. The covariation analysis between antagonistic M1s ranged from short and broad crowns to tall and narrow crowns. The short-crowned upper M1 featuring an expanded hypocone relative to the trigon, covaried with a lower M1 showing a mesio-distal and bucco-lingual expansion of the base relative to the occlusal aspect. The occlusal aspect of the lower M1 seemed less variable than the upper M1, showing more rounded mesial and distal aspects in the relatively broader crowns.

We detected a coherent pattern in the overall crown shape variation of all postcanine tooth types, namely between tall and narrow-crowned versus short and broad-crowned teeth (cf. ‘shape-types’ described by Pfneiszl^46^. A thorough description of the 3D shape changes associated with covariation in the occluding and neighboring tooth type pairs can be found in Fig. 2 and in the Supplementary Information (Supplementary Fig. S1-S15).

## Shape covariation of the EDJ occlusal surface

When focusing on the EDJ occlusal surface dataset alone (excluding information about crown base and height), most of the pairwise correlation values decreased notably (see Supplementary Tables 4 and 5). The pairwise correlation values ranged from r_1_ = 0.41 to r_1_ = 0.80. The highest correlations were observed between upper P3 and lower M1 (r_1_ = 0.80), upper P3 and lower M2 (r_1_ = 0.76), lower P3 and M1 (r_1_ = 0.72), lower M1 and M2 (r_1_ = 0.72), as well as upper M1 and M2 (r_1_ = 0.71). The lowest values were observed between upper M2 and lower P3 (r_1_ = 0.46) and both lower P4 (r_1_ = 0.41) and upper P4 (r_1_ = 0.43). However, the analysis of EDJ occlusal surface shape covariation patterns between various tooth types did not reveal any additional relevant information (see Supplementary Information).

## Geographical variation

Based on our sample, we found a large overlap between populations in shape space (e.g., Fig. 2). A partial separation between some populations was noted in some analyses (see Supplementary Information). However, these instances occurred in the analyses with the most limited sample sizes, including the smallest subsamples (see Supplementary Information), thus we interpret these outcomes as an artefact of the small sample size. However, these findings may be partially driven by morphological tendencies within groups.

## Discussion

In this study, shape covariation of the upper and lower postcanine dentition was explored using a 3D landmark-based approach. According to our results, the dentine crown relative proportions represent the main shape covariation pattern within the postcanine dentition, varying between tall and narrow and short and broad shape-types (Fig. 2). This aligns with the findings of previous work exploring shape variation within geographically diverse samples of different tooth types in modern humans^6,8,13,14,47^. These findings were further supported by the covariation analysis of the sole EDJ occlusal surface, as we observed a decrease in pairwise correlation values throughout the sample when the crown height and base shape were excluded from the analysis. Our results thus reinforce the results of Pfneiszl^46^, who showed that 87% of individuals from a geographically diverse sample tend to have either taller and narrower, or shorter and broader crowns (assessed according to the calculated ST-value of each tooth) across their entire postcanine dentition.

After the elimination of allometric effects from the analyses, the majority of the results remained unchanged, showing that the observed covariation patterns were not driven by size. In fact, we observed only a very small decrease in median value among correlation values derived from multivariate residuals compared to Procrustes coordinates (from 0.74 to 0.70). Since the same morphological covariation pattern reflecting mostly dentine crown proportions was found throughout the whole sample, regardless of the allometric effects, we interpret this covariation pattern as suggestive of morphological integration. This is also in agreement with Pfneiszl’s^46^ findings that specimens showing extreme expressions of either shape-types do not differ in their dentine crown volumes. The gross morphology of the crown is primarily determined by genetic factors^48–50^, although tooth form also reflects functional adaptation. Crown height and proportions play a crucial role in achieving morphological, as well as functional, integration. Our results support this assumption, as we found higher pairwise correlations between tooth types within the same dental row (Table 1). Still, allometric effect is a part of the biological information explaining morphological integration patterns in human dentition to contribute to stable occlusion and effective mastication, even though it only explains a small portion of morphological variation in the modern human postcanine dentition.

In previous studies, high pairwise correlation between the third and fourth premolars was reported for both the lower jaw (r_1_ = 0.87^6^), and the upper jaw (r_1_ = 0.83^14^), with the crown shape-type extreme being either tall and narrow or short and broad. Furthermore, high genetic correlations have been reported between tooth size measurements of upper P3s and P4s for mice and monkeys (length *r* = 0.84; width *r* = 0.92^41^). The present study, considering larger samples including additional human populations, is in agreement with the findings of these studies.

Conversely, pairwise correlations between antagonistic upper and lower P3s and P4s were lower than expected, as we anticipated obtaining very high values reflecting functional modularity mechanisms. This could be explained by the significant variability that lower premolars exhibit on the lingual crown side, owing to differences in their relative mesial and distal fossae expansion, affecting their lingual cusp position^6^. Hence, the non-occluding lingual cusp is free to vary, whereas the buccal aspect is developmentally more stable^51^. Instead, upper premolars show lower morphometric variability of the dentine crown^14,45,52^. A pilot study by Buchegger et al^53^. found a relatively high correlation (r_1_ = 0.76) between lower P3s and upper P4s, which is surprising because these teeth do not articulate in normal occlusion. Yet our results confirm these findings.

Interestingly, upper and lower P4s covaried more closely with the respective M1s rather than with each other. One possible explanation is that this pattern reflects stronger genetic influence and developmental effects, including shared constraints on tooth shape, rather than functional integration. However, premolars are transitional between the anterior teeth and the molars and serve distinct functions. In normal occlusion, the buccal cusp of the lower P3 slides across the interproximal embrasure of the upper canine and third premolar, leading Kraus et al^45^. to consider canines and P3s a “functional entity”. Unlike P4s, P3s participate in tearing rather than crushing or grinding food. Differently, P4’s molarized shape suggests participation in the crashing and grinding activities comparable to molars, highlighting the role of within jaw functional integration in the interplay of postcanine teeth. Hlusko and Mahaney^19^and Hlusko et al^41^. discovered sub-modularity in baboon maxillary post-canine dentitions, with significant genetic correlations between premolar and molar sizes, and higher correlations within each tooth class. Our analysis of shape covariation between human maxillary premolars and molars yielded comparable results, with similar correlation values between upper P4 and both upper M1 (r_1_ = 0.77) and upper M2 (r_1_ = 0.78). Given their distinct functional roles, it is not unexpected to find a lower pairwise correlation between upper P3s and molars. Still, upper premolars show comparable shape covariation patterns with upper molars. This is not surprising, since upper molars feature similar morphologies and correlate highly with each other (r_1_ = 0.83). In the mandibular postcanine dentition, we observed lower correlation values within the molars (r_1_ = 0.73) than between P4s and M1s (r_1_ = 0.79). In general, this high pairwise correlation between P4s and M1s in both upper and lower dental arches is consistent with certain aspects of the field theory expressed by Butler^34^, who claimed that all teeth within the postcanine dentition derive from one type of tooth germ under the influence of incomplete pleiotropy and different effects of morphogens on various tooth types. The hierarchical pattern in which P3 correlates strongly with M1, but not as strongly as with the P4, fits into Butler’s theory on viewing premolars as the mesial part of the molar morphogenic field. However, it is important to keep in mind that this model was established primarily based on phenotypic data, with inconclusive results in subsequent studies aimed at testing Butler´s hypothesis^54,55^. On the other hand, our results are consistent with prior mentioned literature, as the strong within-premolar and within-molar covariation, especially in upper dentition, coupled with somewhat weaker but still substantial covariation between premolars and molars matches published patterns of postcanine covariation and modular organization in mammals and hominins^56^. Our data indicates a hierarchical pattern of morphological integration where correlations are generally stronger within the same dental arch than between antagonists. As this does not change after allometric correction, we do not consider this hierarchy a byproduct of overall size scaling.

High genetic correlation values were reported between upper M1s and M2s in a baboon sample (length *r* = 0.97; mesial width *r* = 0.88; distal width *r* = 0.76), agreeing with our results for shape correlation in human upper molars^41^. In their preliminary study on first molars, Halász^57^ observed a high morphological correlation between the occlusal EDJ surfaces of the upper and lower M1s (r_1_ = 0.85), but a much lower correlation when considering the whole dentine crown (r_1_ = 0.61). Since the pairwise correlation between the upper and lower first molars’ dentine crowns was the highest observed in our study (r_1_ = 0.90), we cannot replicate their results in the current study on extended samples. Very high correlation between antagonistic first molars has been described in the 2D morphological covariation study of Gómez-Robles and Polly^42^, and attributed to the important structural role of first molars as the first permanent teeth to erupt, highlighting the morphological stasis of the upper M1. Morita et al^10^. demonstrated that M1s exhibit the least size variation among upper molars and considered them the most developmentally and morphologically stable tooth type of postcanine dentition, implying that M1s retain the most conservative odontogenetic blueprint of all molars. In the field of dental medicine, first molars are considered pivotal in maintaining the stability of the dental arches throughout growth and development. They play a crucial role in distributing masticatory forces and load, ensuring proper function of the stomatognathic system^58^. This suggests that, apart from possible developmental integration and pleiotropic effects, functional integration might also underlie the selection of certain trait combinations (correlational selection^22^. Our results on the 3D morphological covariation of the upper M1’s dentine crown with other tooth types cannot be used to fully explain the distinct status of the upper M1s within the postcanine dentition. The data shows that upper M1s correlate highly and stably with their neighboring teeth and show very high correlations with all occluding teeth, which distinguishes upper M1s from the rest of the postcanine dentition. However, the high correlation with lower premolars (especially P3) seems to be driven by allometry to a larger extant than in other tooth pairs.

Although we did not investigate the inhibitory cascade model^10,36^, that can be used to explain morphological reduction of distal molars relative to M1, resulting from an increased inhibition versus activation^42^, we cannot support Morita and colleagues’^10^ findings. They report strong allometric effects driving the morphology of upper distal molars, but when projected on the morphological covariation patterns between upper molars, our results show a very high significant morphological correlation between upper M1 and M2 that is not driven by allometry. As Morita et al. did not study upper molars within the same individual’s dentition, this difference might be a byproduct of sampling strategy. However, our results do not explain metameric variation within upper molars but demonstrate that very high morphological covariation between upper molars is reached independently of the size effect on their morphology. Further research is essential to determine whether functional modularity influenced the evolution of the upper M1, enabling it to serve as a key structural pillar of the modern humans’ postcanine dentition.

In contrast to the above-mentioned allometric effect on the morphological covariation patterns between upper M1 and lower premolars, the analyses of lower premolars with upper M2 revealed very surprising results: very high correlations reflecting the shape change in dental proportions that appears to be rather independent of allometric effects. As these teeth are non-occluding and non-adjacent, it is difficult to biologically explain these strong connections. Since the samples for these analyses contain several more African and South American individuals then those for lower premolar vs. upper M1, this deficit should be equalized in future analyses. However, as the samples include mostly the same individuals, the population structure seems less likely to be the reason for this result. On the other hand, since the EDJ occlusal surface of lower premolars does not necessarily have to match upper molars morphologically, the overall proportions of the crown may instead reflect a broader integration pattern across the postcanine dentition. Moreover, a very opposite result was found within the lower jaw between lower premolars and the lower M2, showing that the observed correlation was mainly driven by allometric effect, featuring the greatest decrease in correlation value when analyzing multivariate residuals relative to Procrustes coordinates, throughout the sample. These completely opposing results could be driven by the nature of variation in M2. Both upper and lower M2s are less canalized teeth^42^, and thus show a higher degree of morphological variation, which however, follows a consistent pattern in upper M2 while being more random in lower M2. Hence, observed pairwise correlations, while not fully understood, might reflect a combination of developmental integration, sample structure and the particular nature of M2 variation. Further investigation of this phenomenon is required to understand if this correlation explains a strong signal for a consistent pattern of variation between two unrelated and variable teeth.

Conversely, covariation analyses of upper premolars and lower molars showed moderate correlation values, seemingly not affected by allometry. However, these results don’t show statistical significance and are rather inconclusive according to the Z-values. We assume that these results might be connected to the lower sample size and must be examined again in the future using a larger sample.

A structural constraint of the current dataset is that geographical population background and sex of the individuals remain formally unmodeled. However, empirical Procrustes ANOVAs on a representative tooth class (upper P3) demonstrate that sexual dimorphism has a negligible effect (R^2^ = 0.025, *p* = 0.806) on crown shape. Regarding geographic origin the analysis detected a minor but significant population effect (R^2^ = 0.169, *p* = < 0.001). Post-hoc pairwise testing reveals this is driven primarily by a directional shift in the South American mean shape. However, we acknowledge that this represents a sampling limitation rather than an absolute geographic barrier, as our South American sub-sample consists predominantly of a single group. This restricted internal variation might artificially amplify its distinctiveness in a distance-based ANOVA framework. As demonstrated by previous dental literature^6,8,13,14,47^, human dental variation is inherently continuous, and thus expanding the sample size and incorporating a denser array of global populations serves to fill the intermediate shape space, systematically increasing morphological overlap. Because the majority of shape variance (83.1%) remains rooted at the individual level, these regional cohorts exist within a shared distribution. Ultimately, capturing this geographic heterogeneity ensures that the morphological integration patterns isolated by our 2B-PLS analyses reflect robust covariation pathways operating independently of population backgrounds.These findings provide insight into the evolutionary patterns of the human postcanine dentition. Strong covariation among certain tooth types, combined with the stable and very strong covariation of first molars, aligns with the hypothesis that developmental and functional integration have shaped dental evolution, potentially constraining some traits while allowing others to vary. These results offer a framework for understanding how morphological variation arises and is coordinated across teeth, contributing to our understanding of the driving evolutionary processes in human dental diversity and providing context for both modern and fossil populations. Our study thus lays the morphological groundwork for further research on genotype-phenotype associations for exploring the odontogenetic processes underlying dental evolution in mammals, including the human species.

## Conclusions

This study deepens our understanding of the morphological covariation of human postcanine dentition by analyzing 3D dentine crown shapes across a geographically diverse sample of modern humans. Our results reveal a hierarchical pattern of morphological covariation with high developmental integration within dental arches, especially in the upper jaw, that is stronger than compatibility between antagonistic teeth. This hierarchy was largely preserved after size correcting analyses, with overall tooth shape and crown proportions predominantly influencing the observed covariation patterns, especially in tooth types with high morphological variability, such as the lower premolars. Furthermore, according to the results upper first molar might be an important element for the understanding of postcanine covariation patterns, but its distinct status cannot be fully explained by our data. These results are consistent with the hypothesis of complex genetic-developmental interplay in shaping dental morphology, as proposed by studies identifying heritable modules in postcanine dentition. However, our findings also highlight the need for further investigation into this complex topic, as methodological limitations and uncertainties remain. Future studies should address these issues and refine our understanding of the mechanisms driving shape covariation of the postcanine dentition to contribute to a more comprehensive understanding of human dental evolution.

## Materials and methods

This study investigated the shape covariation among 526 teeth of 136 individuals from seven geographically different human groups (Egyptians, *n* = 26; Europeans, *n* = 123; Near Easterners, *n* = 69; Oceanians, *n* = 59; Sub-Saharan Africans, *n* = 109; South Americans, *n* = 83; Southeast Asians, *n* = 57). In particular, we considered teeth from eight different tooth types, namely 70 lower P3s, 74 lower P4s, 76 upper P3s, 74 upper P4s, 49 lower M1s, 48 lower M2s, 59 upper M1s, and 76 upper M2s. Third molars were not included in this study because their pronounced morphological variability and frequent structural reduction^59^, or even complete absence, hinder reliable assessment of the homology required for geometric morphometric analyses^60^. As our objective was to examine morphological covariation among tooth types rather than to maximize overall dental variability, focusing on more developmentally stable teeth ensured methodological consistency. We relied on the study of osteological specimens from museum collections since they present natural dental wear, rarely show caries, and mostly no dental intervention such as fillings. Importantly, our study required µCT images which cannot be obtained from living subjects. Teeth were selected based on their condition and preservation, preferring individuals presenting as many dental types as possible. We focused on the EDJ which is the primary developmental structure of the crown and consequently the precursor of the enamel cap’s morphology, and is more likely preserved than the outer enamel surface^1,8,61,62^. However, assembling a sample with fully preserved postcanine dentitions from osteological collections was challenging because of missing, broken or severely worn teeth, resulting in differing subsample sizes for the various tooth types. This is mostly the case for upper and lower M1s that are the first postcanine teeth to erupt and thus face a longer period of abrasion and show heavy wear in most adult individuals.

This study builds upon previous projects exploring 3D morphological variation of the EDJ on modern human samples diverse in geographical origin, sex and age^13,6,64,47,57^. A geographically diverse sample was used to maximize the phenotypic variance within the dataset that is essential to avoid underestimating shape variation, as well as for the stability of statistical analyses. By restricting the range of morphological diversity, correlations might even appear artificially low due to limited sample variation^63^. For these reasons, we do not consider the geographical heterogeneity of our samples a possible limitation of this study, but on contrary, a positive aspect.

Nevertheless, a dataset with unmodeled geographical population background and sex cohort, may represent a structural constraint. To evaluate the potential confounding effects of geographical origin and sexual dimorphism on crown morphology, exploratory Procrustes ANOVAs and post-hoc pairwise permutation tests were conducted on raw landmark coordinates of a representative tooth class (upper P3) using R.

### Scanning and segmentation

All specimens were scanned at the Vienna µCT Lab in Austria using an industrial Viscom X8060 NDT scanner with parameters of 110–140 kV, 280–410 mA, 1400–2000 ms, and a 0.75 mm copper filter, achieving a voxel size between 20 and 50 μm. Virtual segmentation of the µCT data was conducted using Amira software (www.thermofisher.com*)* to separate the dental tissues from each other and from the surrounding area, and subsequently generate triangulated surface models of the enamel and dentine. The models were derived from both the right and left side, but the datasets representing right teeth were flipped prior to surface extraction to avoid later reflection of the landmark coordinates. Teeth are not expected to exhibit directional asymmetry^64^; thus, we considered this procedure uninfluential on the outcomes of our study. In cases of slightly worn dentine horn tips (maximal stage 3; small dentine patches^65^, virtual reconstruction was performed in Amira using the ‘brush’ tool by extending the existing contours of the dentine horn tips. Although this method may introduce some degree of subjectivity, it impacts the very tip of dentine horns only, the shape of which doesn’t drive the main shape changes in postcanine teeth^6,64,47,57^. Thus, the impact on morphological covariation patterns is minimal.

### Data collection

The segmented 3D surface models were reoriented in Geomagic Design X 64 (www.3dsystems.com*)* before data collection, following established protocols for the different dental types. The cervical plane, calculated as the best-fit plane to the cervical margin, was used to separate the crown from the roots, and to reorient the teeth parallel to the x-y plane of the virtual workspace. The crowns were then rotated to align the upper premolars’ and molars’ mesial occlusal ridge parallel with the y-axis, the lower premolars’ buccal aspect of the marginal ridge with the x-axis and the lower molars’ lingual aspect of the cervical outline with the x-axis. The outlines of the reoriented cervical surfaces were collected and projected onto the respective cervical planes. The cervical outlines thus represent the contour of the crown at the intersection with the cervical plane^2,66^. These outlines were imported into Rhinoceros 6 (www.rhino3d.com*)* and divided into 24 segments using equiangular (15°) radial vectors originating from the area centroid of each outline. Subsequently, the intersections between the outline and the radial vectors resulted in 24 pseudo-landmarks, representing the interpolated cervical outline of every tooth type. Unlike a standard Procrustes superimposition, we aligned the teeth using morphological features prior to placing the landmarks. This ensures that all landmarks are morphologically homologous, therefore they were not slid but treated as fixed LMs.

### Landmark collection on the EDJ occlusal surface

**Fig. 3:**
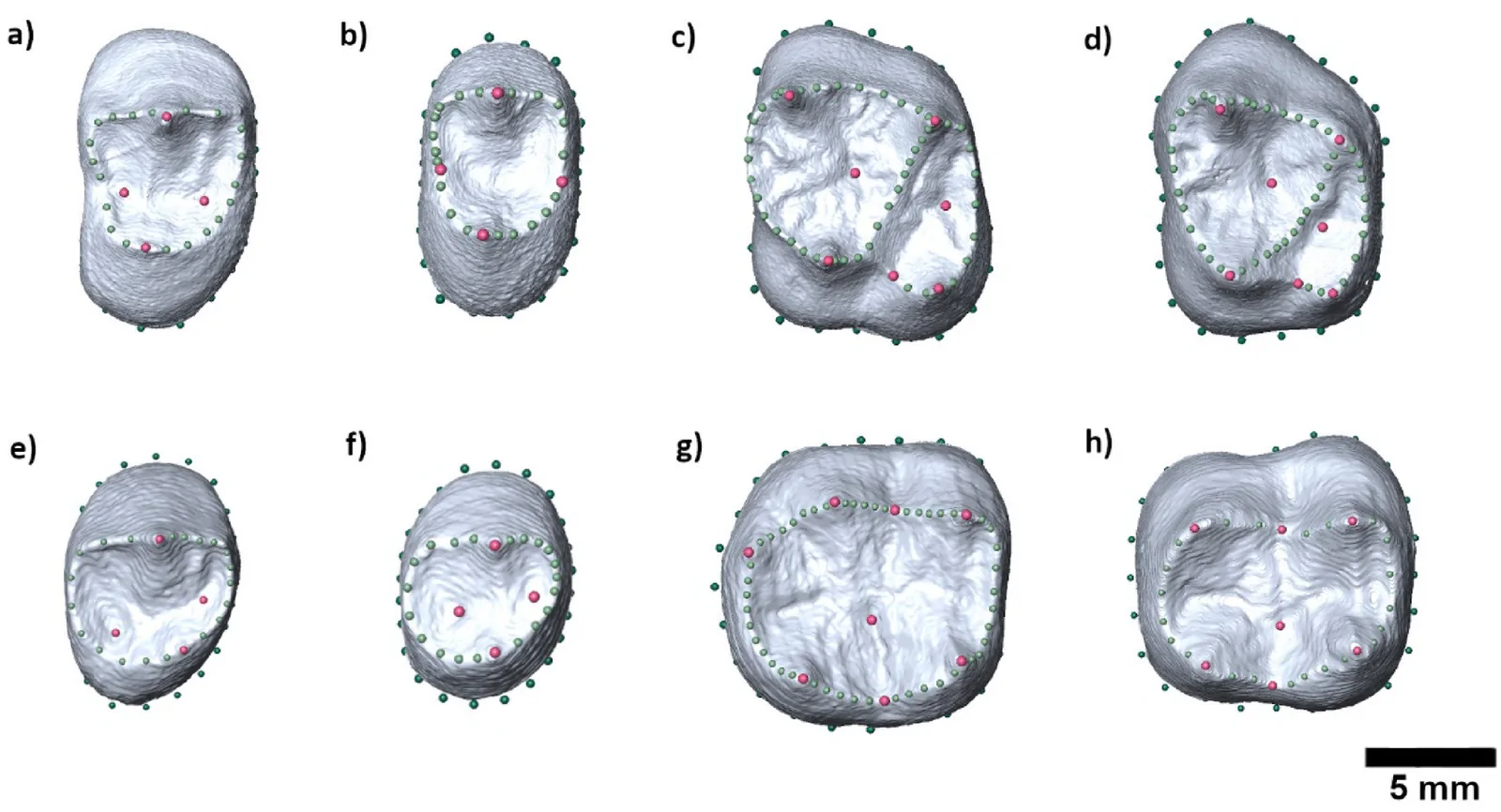
Landmark configurations for the various dentine crown types. Upper third (**a**) and fourth (**b**) premolars, upper first (**c**) and second (**d**) molars, lower third (**e**) and fourth (**f**) premolars, and lower first (**g**) and second (**h**) molars. Fixed landmarks = pink; semilandmarks = light green; pseudo-landmarks = dark green.

The reoriented 3D surfaces of the dentine crowns were imported into the EVAN-Toolbox software (www.evan-society.org) for landmark collection, using configurations suitable to the different dental types (Fig. 3). For all premolars, two fixed landmarks (LMs) were placed on the horn tips of the two main cusps. On both lower premolars^6^, as well as, on the upper P3, two additional LMs were placed at the deepest points of the mesial and distal fossae. Instead in upper P4, where the fossae are less defined, the landmarks were placed on the deepest points of the distal and mesial ridges^5,14^. Additionally, 20 curve semilandmarks (sLMs) were placed along the marginal ridge.

Upper molars were represented by 4 LMs on each of the main horn tips, and 3 additional LMs were placed at the deepest points of the central and distal fossae and on the lowest point between the hypocone and the protocone along the marginal ridge of the EDJ^5^. In both upper molars, 43 sLMs were used to resample the EDJ marginal ridge.

On lower molars, LMs were placed on the tips of the main horns, including the hypoconulid in the LM1, as well as on the lowest point of the lingual and buccal marginal ridge, and the deepest point of the central fossa^15^. The LM1’s and LM2’s marginal occlusal ridges were represented by 47 and 22 sLMs, respectively.

Landmark placement, as well as virtual reconstruction of surfaces may introduce a minimal measurement error. Inter- and intra- observer error has been previously analyzed for these methods using Procrustes distances between observations, revealing a negligible average error of 0.024 and 0.006 units^6^.

### Geometric morphometric analyses

Shape covariation between all postcanine tooth pairs within and between jaws was analyzed in Euclidean shape space using R (R Core Team 2026) and EVAN-Toolbox 1.75 software (www.evan-society.org) using alternatively landmark configurations representing the dentine crowns and those representing the EDJ occlusal surface only. For each of the pairs, General Procrustes analysis^67^ was first conducted to normalize the landmark configurations for each of the dental types. Allometric effects were tested in the lower M2 sample using multivariate regression, including evaluation of various M2 sub-samples. Since allometric effects remain very weak regardless of the composition of the sample, we did not perform this analysis on the remaining tooth types, for which there are published results in the literature^6,64,47,57^. The obtained Procrustes shape coordinates, as well as the multivariate residuals of a Procrustes regression of dentine crown shape on size (represented by natural logarithm of Centroid Sizes), were then analyzed using a 2-block Partial Least Squares (2B-PLS) analysis. In this study, PLS Mode A with symmetric relation between blocks^68^ was employed, as the interest of this study lies in shared covariation between tooth types. Since the accuracy of the RV coefficient is positively affected by the sample size and negatively affected by the number of variables considered^69^, we present the pairwise correlation values (r_1_) that is not as heavily influenced by the ratio of variables to the number of specimens and provides a robust basis for calculating standardized effect sizes (Z-value). For this reason, it makes a more solid descriptor of the strength of integration in high-dimensional shape data providing standardized and easy-to-compare measures^70,71^.

To assess the statistical significance of the pairwise correlations, we have conducted permutation tests with 10,000 iterations, applied to the r_1_ of every tooth pair. Because the dentition represents a highly integrated morphological system, the statistical comparisons are not fully independent. Under such conditions, highly conservative corrections for multiple comparisons may substantially reduce statistical power and increase the risk of Type II errors. We therefore emphasize standardized effect sizes and the broader biological consistency of the observed patterns rather than relying exclusively on adjusted significance thresholds. Additionally, we also report the total squared covariances.

### Ethics statement

We used non-invasive micro-CT scans (a three-dimensional X-ray method) from human remains belonging to collections of the Department of Evolutionary Anthropology, University of Vienna and to the Natural History Museum Vienna, Austria. No ethical committee approval or guidance was required according to the Ethics committee guidelines of the University of Vienna, as the research did not involve present-day human samples (see Supplementary information for further clarification). The remains were exported to Europe between the late 19th century and the beginning of the 20th century. Since these individuals had passed away years or even decades prior to their collection, more than five generations —over 125 years —separate their death from the present study. This temporal distance makes it practically impossible to establish a direct genealogical link to their living descendants. The human remains have been thereafter curated in Austrian institutions for more than a century and were regarded and treated as precious parts of historic collections. The material was handled only by highly trained personnel in the most respectful way. No damage or alterations of any kind were caused to the remains. Given the sensitivity of this matter, we lay open how we handled these data and steps undertaken in regard to the communities of origin.

In this study, individuals from four populations with colonial histories that have developed critical perspectives and corresponding ethical guidelines for research involving their communities were used, namely First People of Australia, Māori from New Zealand, Selk’nam from Tierra del Fuego, and Khoesan from Southern Africa.

The skeletal collections of deceased First People of Australia and Māori from New Zealand have been repatriated, entrusted to the local authorities (in case of the Māori, this included religious rituals taking place in Vienna, Austria), and de-identified for the research conducted on the non-invasive micro-CT scans obtained previously to their repatriation, for the use of which we obtained a written consent. As instructed by authorities of the Department of Communications and the Arts in Canberra of the Australian Government, we avoid publication of visual depictions of the remains.

The skeletal collection of the people of Selk’nam from Tierra del Fuego is currently under an ongoing repatriation process, housed under embargo in the Natural History Museum of Vienna. After virtual consultation with the local representative of the Fundación Hach Saye, informed consent was obtained to use the image data prior to the repatriation.

The skeletal collection of Khoesan individuals is currently under provenance research. As the San Code of Research Ethics (2017) only applies to research involving living people, we have contacted the representatives numerous times by mail, as well as, electronic e-mail, and also reached out in person to various Khoesan populations, as well as the authorities in Botswana and Namibia. Unfortunately, it has not been possible to obtain official consent, as the affinity of the individuals to present-day Khoesan groups is unknown, and therefore they are not considered local to the members of the groups. Still, we keep the individuals de-identified and do not show any depictions of the remains, out of respect towards the deceased individuals.

We are deeply grateful to the Communities of Origin and their representatives that have granted us permission to study the data collected from the human remains of their ancestors, thereby allowing us to contribute to the progress of knowledge in the field of biological anthropology and to the collaboration between nations, institutions, and people. Moreover, we consider it our responsibility to inform descendant communities, whenever possible, about the results of our research, to provide them with our publications, and to remain open to sharing additional information and data with them.

## Supplementary Information

Below is the link to the electronic supplementary material.

Supplementary Material 1

## Data Availability

The data used in this study is not freely accessible, since the authors do not have permission to share these data. The usage of some of the data has been personally requested from third parties and the consent has only been acquired for our group project, without any special access privileges. Consent has to be personally requested and obtained again for every group/researcher that meets the criteria of the third party. The image datasets are stored within the image archive of the Department of Evolutionary Anthropology of the University of Vienna (Anthropologie@univie.ac.at; +43-1-4277-54777; +43-1-4277-54721) and can be requested and accessed with a valid consent from the third party, in the same manner as the authors.
