# Supplementary material for: 3D geometric morphometric analysis of morphological covariation in the modern human postcanine dentition
Source: Sci Rep. 2026 Jun 9;16:26218. doi: 10.1038/s41598-026-56919-2 (PMC13494033; doi:10.1038/s41598-026-56919-2)
Supplement: Supplementary file 1 [file 41598_2026_56919_MOESM1_ESM.docx]

# **Supplementary Information**

# 3D geometric morphometric analysis of morphological covariation in the modern human postcanine dentition

**Petra G. Šimková^1,2*^, Viktoria A. Krenn^1,3,4^, Cinzia Fornai^1,2,3,5,6^, Lisa Wurm^7^, Vanda Halász^8^, Dominika Lidinsky^9^, Gerhard W. Weber^1,2,10^**

^1^Department of Evolutionary Anthropology, University of Vienna, Austria, ^2^Human Evolution and Archaeological Sciences HEAS, University of Vienna, Austria, ^3^Institute of Evolutionary Medicine, University of Zurich, Switzerland,  ^4^Fraunhofer Austria Research GmbH, Klagenfurt, Austria, ^5^Department of Research in Occlusion Medicine, Vienna School of Interdisciplinary Dentistry – VieSID, Klosterneuburg, Austria, ^6^Center for Clinical Research, University Clinic of Dentistry Vienna, Medical University of Vienna, Austria, ^7^Medical Technology Cluster, Business Upper Austria – OÖ Wirtschaftsagentur GmbH, Linz, Austria, ^8^LearnChamp Consulting GmbH & Co KG, Vienna, Austria, ^9^Sandoz GmbH, Kundl, Austria, ^10^Core Facility for Micro-Computed Tomography, University of Vienna, Austria.

**PLS analysis: detailed results**

We performed 2-block Partial Least Squares (2B-PLS) analyses (PLS Mode A; relation between blocks is symmetric) to assess the covariation between tooth types of the human postcanine dentition. A general covariation pattern between tooth types was observed: the dentinal crowns varied between tall and narrow and short and broad.

Despite a general overlap between populations, several analyses revealed partial separations of some geographical groups from some of the remaining groups (e.g. South Americans; Supplementary Fig. S2, S10, S12). Although these separations are most likely driven by small subsample sizes in the analyses, the data nonetheless may indicate certain morphological tendencies within populations. The recent literature on the morphometric analysis of the 3D dentine crown (Halász, 2019; Krenn et al., 2019; Šimková et al., 2021, 2024) led to similar conclusions, showing that distinguishing among populations solely based on their EDJ morphology is not possible, as the overlap between groups increases with increase in sample size.

Here we present 2B-PLS plots for every neighboring or occluding tooth pair. The warps showing the actual shape variation of the teeth are shown in the upper part of the plot for the first, and in the lower part of the plot for the second tooth type.

**A. Morphological covariation of the maxillary teeth**

**Upper P3 – P4**

Upper P3s and P4s covaried between tall-crowned with a narrow base and short-crowned with a mesio-distally and bucco-lingually expanded base. In both tooth types, tall and narrow-crowned teeth presented a mesio-distally slender occlusal aspect with reduced lingual side Conversely, the occlusal aspect of the short and broad crowns was mesio-distally expanded, with a rounded lingual aspect of the EDJ. Additionally, compared to tall-crowned premolars, short-crowned P4s exhibited slightly higher dentinal horn tips relative to the rest of the crown.

**Supplementary Fig. S1** 2B-PLS plot showing the covariation of upper third and fourth premolars’ dentinal crowns; the warping shows the actual shape variation in occlusal and lingual view at the extremities of the range of distribution.

**Upper P4 – M1**

In both, upper P4s and upper M1s, the main shape covariation occurred in the relative expansion of the occlusal area with respect to the base. Both dental types covaried either as narrow-based with expanded occlusal aspect or large-based with a constricted occlusal aspect. Tall and narrow premolar’s buccal part of the occlusal aspect expanded mesio-distally and buccally, with higher buccal cusp relatively to the lingual cusp. The occlusal lingual side was mesio-distally reduced relatively to the buccal side. Along with this premolar shape, the molars showed a relatively expanded trigon and reduced talon, resulting from a bucco-lingual expansion of the mesial marginal ridge and disto-lingually positioned hypocone. With a relative constriction of the occlusal area with bucco-lingually narrower mesial marginal ridge and more disto-lingually bulging hypocone, the premolars expanded lingually.

**Supplementary Fig. S2** 2B-PLS plot showing the covariation of upper fourth premolars’ and first molars’ dentinal crowns; the warping shows the actual shape variation in occlusal and lingual view at the extremities of the range of distribution.

**Upper M1 – M2**

Upper M1s and M2s covaried between tall-crowned with reduced base and short-crowned with expanded base. The tall-crowned upper molars showed relatively expanded trigon and reduced talon with respect to the short-crowned molars, featuring an expansion of the talon and a reduction of the trigon. The differences in the trigon to talon proportions were more pronounced in M1s than in M2s.

**Supplementary Fig. S3** 2B-PLS plot showing the covariation of upper first and second molars’ dentinal crowns; the warping shows the actual shape variation in occlusal and lingual view at the extremities of the range of distribution.

**B. Morphological covariation of the mandibular teeth**

**Lower P3- P4**

Lower P3 and P4 crowns covaried between tall and narrow with mesially shifted lingual cusp and short and broad with relatively expanded mesial fovea, resulting from more centered position of the lingual cusp.

**Supplementary Fig. S4** 2B-PLS plot showing the covariation of lower third and fourth premolars’ dentinal crowns; the warping shows the actual shape variation in occlusal and lingual view at the extremities of the range of distribution.

**Lower P4 – M1**

The analysis of lower P4 and M1 dentinal crowns reflected the joint covariation shape pattern between short and broad crowns, and tall and narrow crowns. The occlusal marginal ridge covaried consistently with the crown base in both tooth types. Tall-crowned P4s featured an increase in height of the buccal cusp in comparison to the short- crowned teeth.

**Supplementary Fig. S5** 2B-PLS plot showing the covariation of lower fourth premolars’ and first molars’ dentinal crowns; the warping shows the actual shape variation in occlusal and lingual view at the extremities of the range of distribution.

**Lower M1 – M2**

The crowns of both lower M1s and M2s covaried between short and broad, with short dentine horns, and tall and bucco-lingually narrower, with taller dentine horns. The bucco-lingual expansion of the occlusal marginal edge matched that of the crown base.

**Supplementary Fig. S6** 2B-PLS plot showing the covariation of lower first and second molars’ dentinal crowns; the warping shows the actual shape variation in occlusal and lingual view at the extremities of the range of distribution.

**C. Morphological covariation between antagonists**

**Upper P3 – Lower P3**

The antagonistic P3s covaried between tall- and narrow-crowned and short- and broad-crowned. Tall and narrow upper and lower P3s showed mesio-distal and disto-lingual reduction of the lingual cusp, with lower P3s additionally showing a relatively taller buccal dentin horn. Short-crowned P3s expanded disto-lingually, with the lower P3s’ lingual cusp shifting more mesially.

**Supplementary Fig. S7** 2B-PLS plot showing the covariation of upper and lower third premolars’ dentinal crowns; the warping shows the actual shape variation in occlusal and lingual view at the extremities of the range of distribution.

**Upper P4 – Lower P4**

Similarly to the P3s, P4s covaried mainly between tall- and narrow-crowned and short- and broad-crowned. The occlusal aspect of the short-crowned P4s followed the mesio-distal expansion of the base, showing a more rounded lingual aspect with respect to the rest of the crown.

**Supplementary Fig. S8** 2B-PLS plot showing the covariation of upper and lower fourth premolars’ dentinal crowns; the warping shows the actual shape variation in occlusal and lingual view at the extremities of the range of distribution.

**Upper P3 – Lower P4**

The teeth covaried mainly from short- and broad-crowned to tall- and narrow-crowned, the former with an expanded distal side of the occlusal aspect creating a rounder shape in both tooth types. Tall- and narrow-crowned lower P4s featured a taller buccal cusp and more centrally placed lingual cusp.

**Supplementary Fig. S9** 2B-PLS plot showing the covariation of upper third and lower fourth premolars’ dentinal crowns; the warping shows the actual shape variation in occlusal and lingual view at the extremities of the range of distribution.

**Upper P4 – Lower M1**

The morphological covariation patterns occurred in the height of the dental crowns and in the expansion of the base relatively to the occlusal aspect. Lingually expanded P4, with mesial shift and buccal inclination of the lingual horn tip, occurred together with the relative inclination of the molar horn tips towards each other, resulting in an elongated occlusal aspect. As the lingual horn tip of the P4 shifted lingually, the horn tips of the molar pointed away from each other. Shorter crowns showed even dentin horns, while taller P4s showed higher buccal dentin horns and M1s higher mesial horns.

**Supplementary Fig. S10** 2B-PLS plot showing the covariation of upper fourth premolars’ and lower first molars’ dentinal crowns; the warping shows the actual shape variation in occlusal and lingual view at the extremities of the range of distribution.

**Upper M1 – Lower M2**

The shape covariation patterns occurred in both tooth types between lower-crowned and square-shaped, and higher-crowned with irregular shaped. This derived from the relative expansion and reduction of the distal and disto-lingual aspect of the dentinal crowns with corresponding bucco-lingual expansion of the mesial side of the crowns.

**Supplementary Fig. S11** 2B-PLS plot showing the covariation of upper first and lower second molars’ dentinal crowns; the warping shows the actual shape variation in occlusal and lingual view at the extremities of the range of distribution.

**Upper M2 – Lower M2**

The antagonistic M2s covaried in height and shape of the crown base. Tall upper M2s were mesio-distally and bucco-ligually constricted, featuring a relatively reduced base in comparison to the occlusal aspect that was more squared, covarying with tall lower M2s showing a bucco-lingually constricted base and expanded distal part of the occlusal aspect. Short-crowned M2s presented a larger base relative to the occlusal aspect, with mesial aspects bucco-lingually expanded. Upper molars showed lingually shifted hypocone, while lower molars’ distal part of the occlusal aspect was bucco-lingually reduced.

**Supplementary Fig. S12** 2B-PLS plot showing the covariation of upper and lower second molars’ dentinal crowns; the warping shows the actual shape variation in occlusal and lingual view at the extremities of the range of distribution.

**D. Morphological covariation between non-occluding tooth types**

**Upper P4 – Lower P3**

The results of the 2B-PLS analysis showed morphological covariation between upper P4s and lower P3s to be mainly related to the general crown proportions. In short crowns, upper premolars with a round occlusal aspect covaried with lower premolars with lingually shifted lingual cusp, resulting in an expanded mesial fossa. Tall-crowned upper P4s featured a mesio-distally narrow occlusal aspect, associated to a bucco-lingual elongation deriving from the lingual position of the lingual cusp, covarying with lower premolars with bucco-lingually reduced occlusal area.

**Supplementary Fig. S13** 2B-PLS plot showing the covariation of lower third and upper fourth premolars’ dentinal crowns; the warping shows the actual shape variation in occlusal and lingual view at the extremities of the range of distribution.

**Upper M1 – Lower P4**

Upper M1s and lower P4s covaried as tall and narrow crowns with a mesio-lingually reduced occlusal aspect in premolars, and disto-lingually in molars, or short and broad crowns with relative expansion of the same aspects.

**Supplementary Fig. S14** 2B-PLS plot showing the covariation of lower fourth premolars’ and upper first molars’ dentinal crowns; the warping shows the actual shape variation in occlusal and lingual view at the extremities of the range of distribution.

**Lower M1 – Upper M2**

Both tooth types covaried as tall crowns with reduced base or short crowns with broad base. The relative proportions of the trigon and talon in upper molars are inverted. The lower molars are expanded distally when the upper molar shows expanded trigon

**Supplementary Fig. S15** 2B-PLS plot showing the covariation of lower first and upper second molars’ dentinal crowns; the warping shows the actual shape variation in occlusal and lingual view at the extremities of the range of distribution.

**E. Pairwise correlation values indicated by occluding/neighboring and non-occluding/non-neighboring status**

| **Supplementary Table S1. Colormap of the pairwise correlation values** resulting from 2-Block Partial Least Squares Analyses. (L = lower; U = upper; P3 = third premolars; P4 = fourth premolars; M1 = first molars; M2 = second molars).  Blue colors indicate occluding or neighboring tooth type pairs; orange colors indicate non-occluding and non-neighboring tooth-type pairs. In both instances, darker colors indicate stronger correlations. | | | | | | | | |
| --- | --- | --- | --- | --- | --- | --- | --- | --- |
|  | LP3 | LP4 | LM1 | LM2 | UP3 | UP4 | UM1 | UM2 |
| LP3 | 1 | 0.89 | 0.76 | 0.74 | 0.71 | 0.75 | 0.77 | 0.88 |
| LP4 |  | 1 | 0.79 | 0.75 | 0.70 | 0.65 | 0.73 | 0.80 |
| LM1 |  |  | 1 | 0.73 | 0.61 | 0.58 | 0.90 | 0.68 |
| LM2 |  |  |  | 1 | 0.63 | 0.66 | 0.82 | 0.64 |
| UP3 |  |  |  |  | 1 | 0.81 | 0.67 | 0.70 |
| UP4 |  |  |  |  |  | 1 | 0.77 | 0.78 |
| UM1 |  |  |  |  |  |  | 1 | 0.83 |
| UM2 |  |  |  |  |  |  |  | 1 |

| **Supplementary Table S2. Percentages of total squared covariance** for the Singular Warp Score 1, resulting from 2-block Partial Least Squares Analyses of the dentin crown datasets. (L = lower; U = upper; P3 = third premolars: P4 = fourth premolars; M1 = first molars; M2 = second molars) | | | | | | | | |
| --- | --- | --- | --- | --- | --- | --- | --- | --- |
|  | LP3 | LP4 | LM1 | LM2 | UP3 | UP4 | UM1 | UM2 |
| LP3 |  | 85 | 86 | 62 | 64 | 67 | 82 | 80 |
| LP4 |  |  | 86 | 66 | 58 | 61 | 74 | 79 |
| LM1 |  |  |  | 60 | 55 | 45 | 70 | 68 |
| LM2 |  |  |  |  | 52 | 58 | 65 | 62 |
| UP3 |  |  |  |  |  | 79 | 46 | 63 |
| UP4 |  |  |  |  |  |  | 59 | 64 |
| UM1 |  |  |  |  |  |  |  | 62 |
| UM2 |  |  |  |  |  |  |  |  |

| Supplementary Table S3. Results of 2-block Partial Least Squares Analyses on Procrustes shape coordinates and on multivariate residuals of a Procrustas regression of shape on size, for every tooth pair. (L = lower; U = upper; P3 = third premolars: P4 = fourth premolars; M1 = first molars; M2 = second molars; r_1_ = pairwise correlation value) | | | | |
| --- | --- | --- | --- | --- |
| Tooth Pair | Type | r_1_ | Effect Size (Z) | *p*-value |
| LP3–LP4 | Coordinates | 0.89 | 5.54 | < 0.001 |
|  | Residuals | 0.90 | 5.13 | 0.001 |
| UP3–UP4 | Coordinates | 0.81 | 5.00 | < 0.001 |
|  | Residuals | 0.82 | 5.15 | 0.001 |
| LM1–LM2 | Coordinates | 0.73 | 2.58 | 0.003 |
|  | Residuals | 0.73 | 2.64 | 0.005 |
| UM1–UM2 | Coordinates | 0.83 | 3.89 | < 0.001 |
|  | Residuals | 0.83 | 3.88 | 0.001 |
| LP4–LM1 | Coordinates | 0.79 | 2.85 | 0.001 |
|  | Residuals | 0.73 | 2.23 | 0.011 |
| UP4–UM1 | Coordinates | 0.77 | 3.06 | < 0.001 |
|  | Residuals | 0.74 | 2.80 | 0.002 |
| LP3–UP3 | Coordinates | 0.71 | 2.74 | 0.003 |
|  | Residuals | 0.70 | 2.63 | 0.002 |
| LP4–UP4 | Coordinates | 0.65 | 2.71 | 0.003 |
|  | Residuals | 0.66 | 2.53 | 0.007 |
| LM1–UM1 | Coordinates | 0.90 | 3.62 | < 0.001 |
|  | Residuals | 0.90 | 4.30 | 0.001 |
| LM2–UM2 | Coordinates | 0.64 | 1.17 | 0.1253 |
|  | Residuals | 0.65 | 1.33 | 0.100 |
| LP3-UP4 | Coordinates | 0.75 | 3.58 | < 0.001 |
|  | Residuals | 0.76 | 3.69 | 0.001 |
| UP3-LP4 | Coordinates | 0.70 | 2.77 | 0.001 |
|  | Residuals | 0.72 | 2.91 | 0.001 |
| LM1-UM2 | Coordinates | 0.68 | 2.06 | 0.0212 |
|  | Residuals | 0.69 | 2.20 | 0.011 |
| UM1-LM2 | Coordinates | 0.82 | 1.92 | 0.025 |
|  | Residuals | 0.84 | 2.28 | 0.009 |
| LP4-UM1 | Coordinates | 0.73 | 2.85 | 0.001 |
|  | Residuals | 0.67 | 2.22 | 0.011 |
| UP4-LM1 | Coordinates | 0.58 | -0.19 | 0.571 |
|  | Residuals | 0.55 | -0.44 | 0.654 |
| LP3-LM1 | Coordinates | 0.76 | 2.80 | 0.001 |
|  | Residuals | 0.72 | 2.57 | 0.005 |
| LP3-LM2 | Coordinates | 0.74 | 2.41 | 0.007 |
|  | Residuals | 0.57 | 0.40 | 0.351 |
| LP4-LM2 | Coordinates | 0.75 | 3.17 | < 0.001 |
|  | Residuals | 0.58 | 1.13 | 0.133 |
| UP3-UM1 | Coordinates | 0.67 | 1.56 | 0.058 |
|  | Residuals | 0.67 | 1.39 | 0.077 |
| UP3-UM2 | Coordinates | 0.70 | 3.17 | < 0.001 |
|  | Residuals | 0.69 | 3.18 | 0.002 |
| UP4-UM2 | Coordinates | 0.78 | 4.18 | < 0.001 |
|  | Residuals | 0.77 | 4.16 | 0.001 |
| LP3-UM1 | Coordinates | 0.77 | 3.02 | < 0.001 |
|  | Residuals | 0.67 | 1.87 | 0.031 |
| LP3-UM2 | Coordinates | 0.88 | 4.88 | < 0.001 |
|  | Residuals | 0.84 | 4.38 | 0.001 |
| LP4-UM2 | Coordinates | 0.80 | 4.08 | < 0.001 |
|  | Residuals | 0.74 | 3.50 | 0.001 |
| UP3-LM1 | Coordinates | 0.61 | 0.10 | 0.454 |
|  | Residuals | 0.60 | 0.07 | 0.482 |
| UP3-LM2 | Coordinates | 0.63 | 0.31 | 0.376 |
|  | Residuals | 0.64 | 0.42 | 0.352 |
| UP4-LM2 | Coordinates | 0.66 | 0.98 | 0.167 |
|  | Residuals | 0.62 | 0.63 | 0.251 |

**F. Morphological covariation of the enamel-dentine junction (EDJ)**

The dominant factor influencing crown shape differences across all tooth types (upper and lower premolars and molars) was consistently the shape-type, resulting in either tall and narrow or short and broad extreme shapes. While the shape-type primarily drives covariation patterns, there is also notable morphological variation on the occlusal surface (EDJ). In some cases, particularly if the compared teeth included an extremely variable tooth type such as lower premolars or lower second molars, the general dominance of the overall crown shape-type may have suppressed the signals of the occlusal aspect. Therefore, we conducted another analysis, excluding the landmark representation of the crown base (cervical outline), thus examining only the EDJ. This led to an overall decrease in pairwise correlation values throughout the sample, resulting in only two high correlation values (upper P3 and lower M1, r_1_ = 0.80; upper P3 and lower M2, r_1_ = 0.76).

We observed recurrent patterns driving morphological covariations between tooth types, such as the relative expansion of the distal aspect (Supplementary Fig. S16 A), the expansion and reduction of mesial and distal aspects relative to each other (Supplementary Fig. S16 B), or joint changes in mesio-distal and bucco-lingual dimensions. On the other hand, some tooth type pairs showed completely unrelated shape changes independently of each other (relative changes in bucco-lingual and mesio-distal dimensions in lower P4s, covaries with relative expansion and reduction of the talon with respect to trigon in upper M2s; Supplementary Fig. S16 C).

However, the joint examination of pairwise correlation values and shape covariation patterns did not reveal any significant information, as the covariation patterns described above did not correspond with, nor were dependent on the strength of the pairwise correlation values.

In conclusion, the EDJ covariation analysis support our findings for the whole dentinal crown analyses, which demonstrated that the large-scale morphology of the teeth, specifically the shape-type, is the primary driver of the covariation within the human postcanine dentition. Relationships between neighboring, antagonistic, and non-occluding/non-neighboring tooth types were, however, not as straightforward as we would have expected. From the existing literature, such expectations could be, for instance, high correlation for neighboring teeth, probably weaker correlation for antagonistic teeth, and lower correlation for non-occluding/non-neighboring teeth. Our findings only partly align with the expected patterns, which is why we believe that this intriguing topic warrants further investigation and exploration.

Given the importance of teeth in many fields of natural sciences and medicine, we have relatively little knowledge of odontogenesis. Aspects of cusp pattern development in the interplay of genetic and epigenetic factors still need to be unraveled. Knowledge about the morphological covariation of the EDJ between and within various tooth classes might help supporting future genetic studies.

| **Supplementary Table S4. Pairwise correlation values** resulting from 2-block Partial Least Squares Analyses of the enamel-dentine junction datasets. (L = lower; U = upper; P3 = third premolars: P4 = fourth premolars; M1 = first molars; M2 = second molars) | | | | | | | | | | | | |
| --- | --- | --- | --- | --- | --- | --- | --- | --- | --- | --- | --- | --- |
|  | LP3 | LP4 | | LM1 | | LM2 | | UP3 | | UP4 | UM1 | UM2 |
| LP3 | 1 | 0.57_(n=60)_ | | 0.72_(n=32)_ | | 0.50_(n=26)_ | | 0.56_(n=39)_ | | 0.50_(n=41)_ | 0.54_(n=35)_ | 0.46_(n=40)_ |
| LP4 |  | 1 | | 0.66_(n=38)_ | | 0.61_(n=32)_ | | 0.60_(n=41)_ | | 0.51_(n=43)_ | 0.52_(n=35)_ | 0.41_(n=40)_ |
| LM1 |  |  | | 1 | | 0.72_(n=32)_ | | 0.80_(n=26)_ | | 0.65_(n=27)_ | 0.67_(n=31)_ | 0.67_(n=33)_ |
| LM2 |  |  | |  | | 1 | | 0.76_(n=22)_ | | 0.65_(n=23)_ | 0.69_(n=21)_ | 0.51_(n=28)_ |
| UP3 |  |  | |  | |  | | 1 | | 0.59_(n=63)_ | 0.67_(n=37)_ | 0.65_(n=51)_ |
| UP4 |  |  | |  | |  | |  | | 1 | 0.61_(n=35)_ | 0.43_(n=50)_ |
| UM1 | |  |  | |  | |  | |  |  | 1 | 0.71_(n=38)_ |
| UM2 | |  |  | |  | |  | |  |  |  | 1 |

| **Supplementary Table S5. Percentages of total squared covariance** for the Singular Warp Score 1, resulting from 2-block Partial Least Squares Analyses of the enamel-dentine junction datasets. (L = lower; U = upper; P3 = third premolars: P4 = fourth premolars; M1 = first molars; M2 = second molars) | | | | | | | | |
| --- | --- | --- | --- | --- | --- | --- | --- | --- |
|  | LP3 | LP4 | LM1 | LM2 | UP3 | UP4 | UM1 | UM2 |
| LP3 |  | 62 | 40 | 35 | 43 | 48 | 39 | 42 |
| LP4 |  |  | 40 | 53 | 54 | 42 | 50 | 50 |
| LM1 |  |  |  | 41 | 44 | 61 | 45 | 46 |
| LM2 |  |  |  |  | 32 | 72 | 46 | 49 |
| UP3 |  |  |  |  |  | 43 | 46 | 49 |
| UP4 |  |  |  |  |  |  | 37 | 51 |
| UM1 |  |  |  |  |  |  |  | 46 |
| UM2 |  |  |  |  |  |  |  |  |

**Supplementary Fig. 16** Thin Plate Spline warps capturing the extreme shapes observed at the negative **(-)** and positive **(+)** ends of the Singular Warp Score 1 in 2B-PLS analyses of the enamel-dentine junctions between **(A)** lower molars, **(B)** upper molar, and **(C)** lower fourth premolar and upper second molar.

**Supplementary information concerning ethics**

This section features additional information and insights into all practices concerning ethics conducted for this research project that are not mentioned in detail in the Ethical statement of the Manuscript.

As the Ethical statement states, no ethical committee approval or guidance was required according to the Ethics Committee guidelines of the University of Vienna, as the research did not involve present-day human samples, and thus is not a subject to the Ethics Committee. These are the Tasks of the Ethics Committee of the University of Vienna, listed in the official Ethical guidelines by the University of Vienna:

‘**Tasks of the Ethics Committee (University of Vienna)**

§ 2. (1) The Ethics Committee issues opinions on the following research projects:

Research projects involving human participants: These are studies that may affect the physical or psychological integrity, the right to privacy, other subjective rights, or overriding interests of the participants. […] (2) In its opinion, the Ethics Committee must assess whether, in carrying out the research project, the protection of the rights, safety, and well-being of the human participants (para. 1 no. 1) […] are adequately ensured.’

Furthermore, we provide detailed information about four populations with colonial histories that have developed critical perspectives and corresponding ethical guidelines for research involving their communities that were used in this study, namely Māori from New Zealand, Selk’nam from Tierra del Fuego, First People of Australia, and Khoesan from Southern Africa.

The human remains of Māori from New Zealand were collected by Andreas Reischek an Austrian traveler, ‘New Zeeland enthusiast’, hunter, taxidermist and autodidact, between the years 1877 and 1889. Reischek safeguarded the human remains until they were finally acquired by the NHM (Eggers et al., 2021), where they were stored until their final repatriation in 2022.

The skeletal remains of Selk’nam individuals from Tierra del Fuego have been collected by German ethnographer and catholic priest Martin Gusinde, and German doctor and anthropologist Robert Lehmann-Nitsche. Gusinde undertook his journey to Tierra del Fuego during which he lived among Selk’nam people between the years 1918 and 1924. He returned to Austria and sold the collection of Selk’nam skeletal remains to the NHM in 1930. Lehmann-Nitsche traveled to Isla Grande de Tierra del Fuego in 1902, where he collected human skeletal remains some of which have been attributed to the Selk’nam population and donated them to the NHM in 1935 (Olivares et al., 2023).

The skeletal remains of First People of Australia were collected by the Austrian anthropologist Rudolf Pöch on his research expedition to Australia and New Guinea from 1904 to 1906. In the following years Rudolf Pöch undertook another expedition (1907 – 1909; Pacher, 1961) during which the remains of some Khoesan individuals were collected by Rudolf Pöch and Rudolf Oldenburg in South Africa, Botswana and Namibia. When he was appointed lecturer for anthropology and ethnography at the University of Vienna in 1910, the human remains were transformed into the first ‘anthropological teaching and research collection’ at that university. In 1919, Pöch became the first professor for anthropology and ethnography in Austria, and founder of the Department of Anthropology at the University of Vienna, where this collection of human remains has been stored since (Matiasek & Wilfing, 2021; Schasiepen, 2021).

For the use of the µCT scans of the human remains of Māori from New Zealand, we obtained a consent during an in-person meeting with the Māori representative Te Herekiekie Haerehuka Herewini in Buchs, Switzerland on the 3^rd^ of November 2025. Te Herekiekie Haerehuka Herewini was present in Vienna during the repatriation of the Māori remains in 2022, as a responsible representative of the Māori people. We obtained official written consent on the 7^th^ of November 2025 (see copy below).

The informed consent to use µCT scans of the human remains of First people of Australia for our research, was sought out already before their repatriation via written correspondence in 2018 with Amanda Morley, the Assistant Director of Museums and Repatriation/ Collections and Cultural Heritage institute of the Department of Communications and the Arts in Canberra, Australia. We received precise guidance in how to proceed in cases of known and unknown group affinity of the ancestral remains. On the 26^th^ of March 2018, Morley wrote the following:

‘*If you wish to consult with the appropriate traditional custodians regarding the information you*

*propose to publish in your study, the Department will assist you with identifying the appropriate*

*community representatives. If you decide to publish the report without seeking prior informed*

*consent, the Department requests that any reference to the Australian Indigenous ancestral remains within your study be de-identified and no visual depictions of the ancestors be published.*

*The Department acknowledges the University’s leadership in repatriation and recognition of*

*Indigenous peoples’ rights to their ancestral remains and associated material. We also note recent discussions to repatriate the remaining Australian Indigenous ancestors still held in your care.*

*If you have any further questions, please contact me directly. I look forward to working with you further to progress these important matters.*

*Yours sincerely*

*Amanda Morley*’

The following research on population affinity of the human remains that had relied on Rudolf Pöch’s written information only (1904-1906), was unfortunately not successful. We were thus compelled to approach the solution of de-identifying any references to the Australian Indigenous ancestral remains.

This was a feasible solution in our case, as we analyzed individuals of various geographical origin within larger continental and regional groups in this study. This would however not be satisfactory for deeper analyses concentrating on Australian Indigenous ancestral remains as the primary interest of a research project, in which case authors would need to find a different solution. We would like to emphasize the latter as a guideline for the authors of possible future projects involving Australian Indigenous ancestral remains.

The informed consent to use µCT scans of the ancestral remains from the Tierra del Fuego collection from the NHM in Vienna for our research purposes was obtained via an online meeting on the 31st of July 2023 by Selk’nam representative Fernanda Olivares. In the subsequent correspondence Olivares wrote the following:

‘*In my opinion, human remains are not just another part of a collection. They are my ancestors and they deserve respect and consideration. It is hard enough that they aren't resting where they belong (Tierra del Fuego), or even complete (most of the human remains collections, at least the ones i've known of, they are mostly craniums, that was the only part important at the time the bones were collected a century ago.*

*When researchers take a part of the bone, they are destroying a tiny part of that ancestor that remained "complete" over the decades. From my perspective, thinking and behaving like a bone is soulless is naive and irresponsable.*

*On the other hand, studying the structure of the bones through virtual scans is more considerate with the ancestor. Once you scan them, a digital image will be a part of the collection and you only have to disturb the ancestor once and every researcher can revisit a digital image any time it's needed.*

*Ideally, all human remains, at least those taken from Tierra del Fuego, they should be resting, buried, and safe. I know that's not possible in a short term, that's why i consider extremely important to practice a indigenous friendly investigation, and having ct scan will definitely allow students and researchers to study what they need, doing no harm to the few parts of my own story that is still far away from home.*

*I'm truly interested in your investigation. You are not focusing specifically in Selk'nam remains (this is nice believe it or not) but in human history. Also, you are in no level disturbing the actual remains, only their pictures and that's also a very nice technique. I've always spoken about contradictions in myself. I love to learn new information, especially when this new information might have something to do with my own story.*

*But the contradiction is that most of the information have to be discovered and to do so, ethnographic and human remains collections must be studied. With your research, i will be able to learn little bit more of how the ancestors you'll use lived and this knoledge can be passed to my people in Chile. I think it's bigger than i can describe.*

*I hope this answers are usefull and if you think you can use anything of what i wrote, of course you can quote me.*

*Looking forward to find time to meet you in Vienna!*

*Saludos*

*Fernanda Olivares*

*Ingeniera en administración hotelera.*

*Directora Ejecutiva*

*Fundación Hach Saye’*

(Fernanda Olivares, Fundación Hach Saye, 17^th^ of August 2023)

The skeletal collection of Khoesan individuals has been carefully curated at the Department of Evolutionary Anthropology, University of Vienna over the last 117 years. It represents one of the largest intact collections of the Khoesan people in the world and was therefore also studied by researchers from South Africa (e.g., Botha & Steyn, 2013). The San Code of Research Ethics (2017, <https://www.globalcodeofconduct.org/affiliated-codes/>), however, applies to research involving living individuals only. Nevertheless, we have contacted the representatives of the Khoesan people numerous times by mail, as well as, electronic e-mail between 2021 and 2023 with no result. In 2025, we reached out locally in person to various Khoesan populations, as well as the authorities in Botswana and Namibia, among them the Ministry of Gender Equality, Poverty Eradication and Social Welfare, Disability Affairs and Marginalized Communities in Windhoek, Namibia and to the chief of a large Khoesan community in Ghanzi, Botswana. Unfortunately, it was not possible to obtain official consent, with the main obstacle being no clear affinity of the human remains to present-day Khoesan groups. As the populations had to move in the last 100 years and are spread over large areas of southern Africa, it is hardly possible to know more about the collected individuals without any genetic analysis. Thus, the affinity of the individuals to present-day Khoesan groups is unknown, and therefore they are not considered local to the members of the Khoesan groups.

We therefore proceed as we were advised, and keep the individuals de-identified. Additionally, we do not show any depictions of the remains, out of respect towards the deceased individuals.

**References**

Botha, D., & Steyn, M. (2013). Khoe-San skeletal collections in Vienna and Paris: Origin, history and context. *South African Archaeological Society Goodwin Series*, *11*, 7–12.

Halász, V. (2019). *Morphological assessment of modern human upper and lower first molars* [Masterthesis]. University of Vienna.

Krenn, V. A., Fornai, C., Wurm, L., Bookstein, F. L., Haeusler, M., & Weber, G. W. (2019). Variation of 3D outer and inner crown morphology in modern human mandibular premolars. *American journal of physical anthropology*, *169*(4), 4.

Pacher, H. M. (1961). Anthropologische Untersuchungen an den Skeletten der Rudolf Pöch'schen Buschmannsammlung. In Ö. A. d. W. Wien (Ed.), Rudolf Pöchs Nachlass (Vol. Serie A: Physische Anthropologie). Kommissionsverlag der Österreichischen Akademie der Wissenschaften.

Šimková, P. G., Weber, G. W., Ramirez Rozzi, F. V., Slimani, L., Sadoine, J., & Fornai, C. (2021). Morphological variation of the deciduous second molars in the Baka Pygmies. *Scientific Reports*, *11*(1), 1. https://doi.org/10.1038/s41598-021-95524-3

Šimková, P. G., Wurm, L., Fornai, C., Krenn, V. A., & Weber, G. W. (2024). Shape variation in modern human upper premolars. *PLOS ONE*, *19*(4), e0301482. https://doi.org/10.1371/journal.pone.0301482
